# Multi-Level Stakeholder Perspectives on Determinants of Point of Care Ultrasound Implementation in a US Academic Medical Center

**DOI:** 10.3390/diagnostics11071172

**Published:** 2021-06-28

**Authors:** Anna M. Maw, Megan A. Morris, Juliana G. Barnard, Juliana Wilson, Russell E. Glasgow, Amy G. Huebschmann, Nilam J. Soni, Michelle Fleshner, John Kaufman, P. Michael Ho

**Affiliations:** 1 Division of Hospital Medicine, University of Colorado, Aurora, CO 80045, USA; michelle.fleshner@cuanschutz.edu (M.F.); john.kaufman@cuanschutz.edu (J.K.); 2 VA Center of Innovation for Veteran Health (COIN), Adult and Child Consortium for Health Outcomes Research and Delivery Science (ACCORDS) University of Colorado School of Medicine, Aurora, CO 80045, USA; megan.a.morris@cuanschutz.edu (M.A.M.); juliana.barnard@cuanschutz.edu (J.G.B.); 3 Department of Emergency Medicine, University of Colorado, Aurora, CO 80045, USA; juliana.wilson@cuanschutz.edu; 4 Dissemination and Implementation Science Program of ACCORDS, Department of Family Medicine, School of Medicine, University of Colorado, Aurora, CO 80045, USA; russell.glasgow@cuanschutz.edu; 5 Division of General Internal Medicine and Center for Women’s, Health Research, Dissemination and Implementation Science Program of ACCORDS, University of Colorado School of Medicine, Aurora, CO 80045, USA; amy.huebschmann@cuanschutz.edu; 6 Division of Pulmonary and Critical Care Medicine and Division of General and Hospital Medicine, University of Texas Health San Antonio, Section of Hospital Medicine, South Texas Veterans Health Care System, San Antonio, TX 78229, USA; sonin@uthscsa.edu; 7 Cardiology Section, Rocky Mountain Regional VA Medical Center, Division of Cardiology and Data Science to Patient Value Program, University of Colorado School of Medicine, Aurora, CO 80045, USA; michael.ho@cuanschutz.edu

**Keywords:** point of care ultrasound, implementation science, adoption

## Abstract

There is growing interest from multiple specialties, including internal medicine, to incorporate diagnostic point of care ultrasound (POCUS) into standard clinical care. However, few internists currently use POCUS. The objective of this study was to understand the current determinants of POCUS adoption at both the health system and clinician level at a U.S. academic medical center from the perspective of multi-level stakeholders. We performed semi-structured interviews of multi-level stakeholders including hospitalists, subspecialists, and hospital leaders at an academic medical center in the U.S. Questions regarding the determinants of POCUS adoption were asked of study participants. Using the framework method, team-based analysis of interview transcripts were guided by the contextual domains of the Practical Robust Implementation and Sustainability Model (PRISM). Thirty-one stakeholders with diverse roles in POCUS adoption were interviewed. Analysis of interviews revealed three overarching themes that stakeholders considered important to adoption by clinicians and health systems: clinical impact, efficiency and cost. Subthemes included two that were deemed essential to high-fidelity implementation: the development of credentialing policies and robust quality assurance processes. These findings identify potential determinants of system and clinician level adoption that may be leveraged to achieve high-fidelity implementation of POCUS applications that result in improved patient outcomes.

## 1. Introduction

Point of care ultrasound (POCUS) is ultrasound imaging that is acquired and interpreted by a clinician at the bedside. Driven by growing clinical evidence [1,2,3], there is increasing interest in the integration of POCUS use into routine clinical care by multiple specialties. Though emergency medicine [4] and critical care [5,6] were the first specialties to integrate POCUS into their training standards, multiple other medical and surgical specialties are following their lead, including internal and hospital medicine whose professional societies now officially endorse diagnostic POCUS use [7,8].

In spite of the growing evidence and interest from the clinical community [9], POCUS use has not yet been implemented into practice broadly. Prior studies indicate the cost of equipment and training opportunities were the most commonly reported barriers to adoption by clinicians [10]. However, recently, the cost of ultrasound equipment has dropped dramatically, allowing handheld ultrasound devices to be purchased directly by clinicians. Additionally, more professional societies have developed courses and training pathways for generalists. While recent surveys have published determinants of clinician adoption of POCUS [11], barriers likely vary significantly by local setting. Therefore, implementation efforts must begin with assessing current determinants experienced by local clinicians as implementation will certainly fail without their buy-in.

The growing interest from clinicians across specialties and their ability to directly purchase personal handheld ultrasound devices has compelled hospitals to consider the need to develop local policies and invest in infrastructure to ensure the security of POCUS images as protected health information and quality assurance of these images. Accordingly, attention has now broadened to include determinants of adoption at the health system level, in order to ensure high-fidelity and sustainable POCUS use [12,13,14,15]. For instance, in January 2020, the Joint Commission endorsed a statement by the Emergency Care Research Institute (ECRI), a medical safety advisory group, that many healthcare facilities do not currently have adequate infrastructure, policies, and processes in place to ensure optimal and safe clinical use of POCUS [16,17]. However, determinants of POCUS adoption at the health system level, including credentialing policies, quality assurance processes, and information technology infrastructure, as well as the value proposition of POCUS implementation from the system perspective, are not well described.

Given that successful implementation of any technology requires adoption at both the clinician and health system level, prior studies that have focused on determinants of clinician adoption are necessary but not sufficient. Determinants of adoption at the system level will need to be addressed in order to facilitate adoption at the clinician level. The purpose of this qualitative study was to understand the current determinants of POCUS adoption at *both* the clinician and system levels in order to identify implementation strategies that could facilitate adoption at academic medical centers.

## 2. Materials and Methods

### 2.1. Study Design

We performed a qualitative study to capture broad perspectives on determinants of POCUS implementation at both the system and clinician levels. Data were collected for this study from semi-structured interviews of stakeholders in diverse professional roles within a hospital system. This study is part of a larger study investigating the implementation of point of care lung ultrasound by hospitalists in the care of hospitalized adults during the COVID-19 pandemic.

### 2.2. Conceptual Framework

Given the multi-level institutional and external factors that affect the adoption of POCUS by clinicians and health systems, we selected the Pragmatic Robust Implementation and Sustainability Model (PRISM) to frame our investigation. PRISM is a pragmatic multi-level contextual model, includes relatively specific domains relevant to POCUS, and is tied to implementation outcomes in the RE-AIM framework [18,19,20]. The RE-AIM [20] framework was developed to promote external validity and equity in research on health interventions and assesses both implementation and effectiveness outcomes. The contextual domains of PRISM include known drivers of implementation [19] in the External environment (i.e., national policies, guidelines, and incentives) and the Internal setting (i.e., multi-level organizational characteristics, perspectives, implementation and sustainability infrastructure). The use of PRISM was recommended for the planning stages of implementation of health interventions to help identify determinants (i.e., barriers and facilitators) that will inform the creation and selection of implementation strategies, thereby enhancing adoption, implementation and maintenance of evidence-based practices [18]. The contextual domains of PRISM were used to guide the interview protocol, data coding, and analysis.

### 2.3. Study Sample and Setting

We interviewed multi-level stakeholders at University Colorado Hospital, a 550-bed quaternary care academic medical center in Aurora, Colorado. Interviews were performed as part of a pilot study investigating lung ultrasound implementation by hospitalists during the COVID-19 pandemic. A key stakeholder was defined as an individual who has an influence on POCUS adoption. Stakeholders included hospitalists, subspecialists, radiographers, administrators, information technologists, clinical and non-clinical hospital leadership. Patients and trainees were not recruited because there is already a body of literature demonstrating both types of individuals support POCUS use and are drivers of adoption [21,22].

This study was approved by the Institutional Review Board of the University of Colorado in April 2020. Postcard consent was obtained from all participants. We used purposeful sampling for initial study recruitment and snowball sampling to complete enrollment.

### 2.4. Data Collection

Between July 2020 and January 2021, two investigators (JGB and AMM) conducted semi-structured interviews with key stakeholders to understand their perspective on POCUS implementation in their local setting and more broadly. We developed interview guides for each stakeholder demographic (Supplementary Material File S1) which were guided by the contextual domains of PRISM and evolved over the course of data collection. Interviews were conducted by phone or video conferencing. Data collection continued until preliminary analyses indicated thematic saturation when no additional themes were emerging from the interviews.

### 2.5. Data Analysis

Interviews were audio-recorded and transcribed verbatim by a professional transcription service. A mixed deductive and inductive coding process aligned with a framework method approach was used [23]. The framework for deductive codes included the contextual domains of PRISM for the external and internal environment (Table 1). We additionally allowed for new codes that inductively arose from the data. Two investigators (AMM and JGB) began the analysis by immersing in the data and then developing the initial coding framework based on the PRISM domains, which was independently applied to a subset of transcripts. The research team then met multiple times to reconcile coding, refining and further developing the coding framework until a final coding framework was agreed upon. One investigator (AMM) applied the framework to the remaining transcripts, with a second investigator (JGB) double coding 20% of the transcripts to ensure consistency of application of the codes across the transcripts. All discrepancies were reconciled through consensus. The codebook and analysis were reviewed by other research team members (MF and JK) and MAM, a doctoral-trained qualitative expert. Coded data were analyzed within and across different stakeholder groups to identify major and minor themes that represent the participants’ perceptions of POCUS implementation determinants.

## 3. Results

Of the 36 stakeholders invited, a total of 31 key hospital stakeholders participated in interviews that lasted 20 to 45 min (Table 2). In addition to hospitalists, seven subspecialty clinicians from cardiology (1), nephrology (1), anesthesia (1), pulmonology/critical care (1), and emergency medicine (3) were interviewed. Recruited clinicians had a broad spectrum of POCUS experience ranging from novice to experts who routinely use POCUS for diagnosis of multiple disease processes including pneumothorax, pneumonia, pleural effusion, and decompensated heart failure. Hospital leaders included quality and safety leaders, clinical operations leaders including radiology informatics, a hospital medicine clinical leader, and a leader from the health system’s POCUS task force committee. Hospital administrators included an administrator for the division of hospital medicine and radiography. Support staff interviewed included two information technologists and a radiology technician. 

Although our data fit well within the contextual domains of PRISM, there were two domains within the external environment in which no data were collected: policy and incentives. No themes regarding external incentives related to POCUS emerged spontaneously from these data, and no question on our interview guide explicitly inquired about this topic, although views regarding clinical practice guidelines were collected which is an alternative domain within the external environment of PRISM, in contrast to policy. 

### 3.1. Themes

Cutting across our PRISM codes and domains, three dominant themes emerged from stakeholder interviews about a system- and clinician-level adoption: clinical impact, efficiency and cost (Table 3). Although these three themes were central in discussions of adoption, the relative importance and relationship between themes differed by stakeholder level. Subthemes also varied slightly by stakeholder level. Hospital leaders generally focused on determinants of system-level adoption and clinicians were focused on determinants at the clinician level. Cost or financial impact was perceived as the fundamental arbiter of system-level adoption with quality metrics and measures of efficiency, such as length of stay, acting as a surrogate for cost. In contrast, although clinician stakeholders acknowledged that high-value practices were desirable, they seemed more concerned with the potential costs to patients as opposed to costs to the hospital. Additionally, clinician stakeholders often placed more emphasis on clinical impact in terms of patient outcomes and personal workflow efficiency with regard to their own likelihood of adoption. Table 4 offers additional quotations supporting the themes discussed.

#### 3.1.1. Theme 1: Clinical Impact

Across stakeholder types, the perception that POCUS use had the potential to both benefit and harm patients was a central theme in the discussion of determinants of both clinician and system adoption. Ensuring adequate training was a subtheme among clinicians when discussing determinants of personal adoption. The creation of hospital policies around POCUS credentialing, privileging and quality assurance infrastructure was a subtheme among hospital leaders. Patient perceptions of POCUS were seen as a determinant of clinician and health system adoption by both clinicians and hospital leaders. Many clinicians who had experience using POCUS commented they perceived it enhanced their rapport with patients and practice experience.

#### 3.1.2. Potential for Both Clinical Benefit and Harm

*Clinician-level adoption:* Many clinician stakeholders reported that they perceived clinical decision-making could be enhanced by POCUS because of the improved accuracy in diagnosing decompensated heart failure, pneumonia, pleural effusion, and pneumothorax, and in doing so, would expedite initiation of appropriate therapies. This perceived benefit to patient care made POCUS adoption attractive to clinician stakeholders across specialties. For instance, clinician J1 said: “*If you’re concerned, you don’t have time to get radiography, and so having access to ultrasound– it’s essential to make the diagnosis*”.

However, many clinician interviewees considered patient harm from POCUS misuse due to inadequate training as an important potential pitfall of POCUS use. Another potential pitfall mentioned was if clinicians apply ultrasound outside the scope of either its intended use of their specialty’s practice. For instance, clinician E2 was quoted as saying “*People using ultrasound in the, quote, ‘wrong way’ …[end up] saying a lot more than they are qualified to say*”. Many clinicians interviewed expressed that access to adequate training and clear guidelines for use were important prerequisites to their personal adoption of POCUS.

*System-level Adoption:* The need to ensure the quality of care with appropriate credentialing and quality assurance mechanisms emerged as important perceived determinants of adoption at the system level. Hospital leader interviewees emphasized the importance of robust quality processes to ensure high-fidelity system-level adoption of POCUS. For instance, when discussing the reasons why the hospital had recently decided to form a POCUS task force committee, hospital leader G7 said it was an attempt to safeguard appropriate use, “*How do we assure that people are utilizing the tool correctly and not tinkering with a diagnostic procedural skill without a clear understanding?*”.

#### 3.1.3. Patient and Physician Experience Related to POCUS Use

*Clinician-level adoption:* Many clinicians reported improved patient–physician rapport and experience with POCUS use as an important advantage of POCUS. The additional clinician time at the bedside required to perform POCUS exams allowed for additional conversation and rapport building between the clinician and patient, as well as augmented history gathering. Clinician A27 stated: “*I think it also increases the amount of time that I’m with patients, and so there’s probably some either measurable or unmeasurable intangible thing there that it builds rapport*”. Clinicians reported reviewing POCUS images with patients as they are acquired in real-time which allowed for enhanced conversations regarding their medical issues, offering an opportunity for patient education.

*System-level Adoption:* The potential impact of POCUS adoption on patient satisfaction scores was perceived by hospital leaders as an important determinant of system-level adoption. Patient satisfaction scores are a reportable metric and could potentially impact the number of patients seeking care, thereby influencing hospital revenue. Hospital leader G6 stated: “*They [hospital executives] care about their rankings so probably reported patient experience scores, and then they care about the brand. I suppose you could make a case that you could market this as, ‘We are technologically innovative in a way that other health systems are not.’ That might be an appeal*”.

#### 3.1.4. Theme 2: Efficiency

*Clinician-level Adoption:* Many stakeholders felt that clinicians had to tolerate a period of reduced efficiency in their personal clinical workflow in order to adopt POCUS, but once the skill was mastered, it would improve the efficiency of their diagnosis and management of patients. For instance, a novice POCUS user may take more time to perform and incorporate a lung ultrasound exam into clinical decision-making compared to traditional approaches using auscultation and chest X-ray. However, as a clinician gains experience, incorporating point of care lung ultrasound was perceived as improving efficiency in diagnosis and management. In the words of clinician A18: “*I think if you’re able to invest that time you actually may come out ahead in terms of providing appropriate care for your patients. It’s kind of like your catch 22*”. Multiple clinician stakeholders observed that to complete the process of adoption, clinicians needed to believe that the required time investment in practice would eventually pay off in more accurate and quicker clinical decisions. 

Many clinicians interviewed believed POCUS increases clinician time at the bedside with the patient. As a result, even clinicians who had integrated POCUS into their daily practice reported performing fewer exams when they were caring for a large number of patients. “*If I’ve got a much higher patient census, more patients I have to take care of, I’m less likely to use it ‘cause I don’t have time*”, Clinician A7 stated. When the patient volume was high clinicians reported relying instead on radiology performed tests such as chest X-rays because they take less clinician time to use as they only require a moment to place the order. However, they also acknowledged that their clinical decision-making was delayed with the use of chest X-rays instead of POCUS as they then had to wait for the technician to acquire the images and radiologists to interpret them.

*System-level adoption:* Increased hospital efficiency was identified by hospital leaders as one of the most important facilitators of system-level adoption for all POCUS applications. Hospital leader G6 said: “*Honestly, my experience is if you want to get anything done in hospitals, you have to go after the efficiency piece*”. Length of stay was considered one of the most important measures of system efficiency by hospital leaders. A decrease in ordering low-yield radiology tests, such as portable chest X-rays, was also considered a potentially valuable benefit of POCUS use. Support Staff D2 said: “*I think POCUS would probably be utilized a little more judiciously [because it’s performed by clinicians], rather than pressing a button and saying, ‘X-ray for 14 days every day while the patient’s here’*”.

#### 3.1.5. Theme 3: Cost

*Clinician-level Adoption:* Many clinician stakeholders reported that providing “high-value care” was important and perceived POCUS use as facilitating high-value care because of its potential to expedite diagnosis, appropriate treatment, and discharge while potentially avoiding the need for additional diagnostic tests, such as chest X-ray. Clinician J1 said: “*For me, I think the idea of cost containment and medicine is huge, especially in the intensive care unit where resource allocation is just so extreme. If you had outcome data to suggest that this [POCUS] is beneficial, and we’re actually containing the cost for the hospitalization, that’s a no brainer then.”* In contrast, some clinicians worried that POCUS utilized unnecessarily would incur an extra expense for patients. Clinician A7 stated: “*A group of hospitalists and providers are concerned that, even though it’s a pretty low-cost test, that if we’re charging patients for a test that isn’t really changing our management at all...then that’s a test we shouldn’t be doing*”.

*System-level Adoption:* Hospital leaders perceived financial impact on the health system as the single most important determinant to system-level adoption, even though fully understanding the financial impact was a complex task. Although patient satisfaction, quality of care, and hospital efficiency were considered important aspects of system-level adoption, their impact on the financial health of a hospital was critical to their importance to the health system. Hospital leader G6 stated: “*… it’s corporate America, and it’s about the bottom line*”.

A concern expressed by hospital leaders was a lack of clarity about who should pay for the implementation of a new intervention, particularly one that involves clinician training such as POCUS. Some stakeholders stated that in academic settings, hospital executives may question whether the hospital and health system should bear the cost of clinician training or whether it should be paid by the medical school or clinicians themselves. Hospital Leader G6 demonstrates this perception with the following quote, “*I think university hospital would say, ‘Wow, that sounds like education. The dean does education. The dean should pay for this’*”.

## 4. Discussion

We sought to understand the current determinants of POCUS adoption at the system and clinician level in a quaternary care academic medical center in the United States. Our results suggest that determinants of adoption at both stakeholder levels are well aligned with cost, efficiency, and clinical impact. Most stakeholders interviewed believe that POCUS has the potential to improve outcomes, patient and clinician experience, efficiency, and cost. However, many study participants emphasized that adequate training, credentialing policies, and quality assurance processes must be implemented to realize these potential benefits and prevent potential patient harm from inappropriate POCUS use. 

The themes and sub-themes that emerged from these data on improving outcomes, patient and clinician experience, and reducing cost, map nicely onto the Quadruple Aim [24,25] that seeks to improve patient outcomes, patient experience/satisfaction, health care system costs, and health care clinician/staff satisfaction. The Quadruple Aim is a framework used by many health care agencies including the Agency for Healthcare Research and Quality to guide optimization of the health care system. The Quadruple Aim framework may be used as a tool in future studies to understand the extent to which the implementation of POCUS can contribute to high-value sustainable care.

There have been many surveys assessing barriers to POCUS adoption from the clinician’s perspective [11,26,27,28,29,30]. This study expands notably upon those data. It is the first qualitative study that explores the determinants of clinician- and system-level adoption from the perspective of both clinical and non-clinical stakeholders in a high-resource health system. Given the call for robust quality assurance procedures in U.S. hospitals [16], POCUS adoption will require changes to health system infrastructure in addition to clinician behavior change. Our study adds to the literature on perceived advantages and barriers to POCUS use at both system and clinician levels. Given the concerns that were raised by clinicians and health system leaders regarding the importance of implementing POCUS in a manner that ensures patient safety and the cautions published by national organizations regarding the need for robust quality assurance processes [16], further evaluation of the determinants of high-fidelity implementation is warranted.

In addition to its potential to improve patient health outcomes, many clinician interviewees perceived a benefit from the enhanced patient–physician experience associated with POCUS use. While improved patient experience associated with POCUS was suggested in the literature previously [21,22], to our knowledge, the positive impact on clinician experience, while described in multiple editorials, has not before been demonstrated in primary data. The impact of POCUS use on clinician experience should be explored and characterized in future studies. This is particularly important given the epidemic of burnout among clinicians and the growing recognition of clinician experience as an underpinning of quality health care [24]. 

In addition to clinical outcomes, we learned that POCUS’s effect on efficiency may be an important determinant of both clinician- and system-level adoption. This emphasis on efficiency is not surprising given the hospitalist movement was born out of a desire to increase the efficiency of hospitals and control costs [31]. Although some evidence is emerging that POCUS use can improve clinician and system efficiency [2,32,33], more studies are needed to understand which applications and in what context POCUS is most likely to provide this benefit. 

Finally, the financial impact of POCUS on the health system and the patient was considered an important determinant to system and clinician adoption by many participants. Hospital leaders as well as some clinicians interviewed considered cost as the most important determinant of adoption at the systems level. Notably, many clinician participants considered high-value care an important aspect of clinical decision-making. The generalizability of this finding should be explored in future studies as the concept of high-value care is relatively new and the extent to which it has been internalized by clinicians broadly is unknown. 

The findings on POCUS adoption by a health system and its clinicians captured in the present study mirror professional society conversations around this topic currently [8,15]. As the evidence for the utility of POCUS has grown with multiple studies demonstrating improved accuracy [34], expedited diagnoses [2], and an associated reduction in additional testing and overall costs [32,33,35], there is now an increasingly recognized need for guidelines in training standards and quality assurance to guide implementation [13,14,16].

From an implementation science perspective, given that high-fidelity implementation of complex health interventions, such as POCUS, are known to be highly context-dependent [36], traditional guidelines that offer generic recommendations will likely be insufficient to ensure the benefits of POCUS are realized locally. Instead, guides may help local interested parties assess determinants of adoption unique to their environment, develop quality assurance infrastructure, and evaluate the effectiveness of local processes. Such adoption (and potentially implementation, adaptation and sustainability) guides can help ensure the potential benefits of POCUS use are reproduced in diverse real-world practice settings. The PRISM contextual model seemed to work well in categorizing the themes that emerged, but we note that not all PRISM domains were discussed and that alternative models of contextual factors related to adoption may also have fit the emergent data such as Rodger’s Diffusion of Innovation Theory [37], Normative Process Theory [38] and Consolidated Framework for Implementation Research [39]. Finally, this study focused on determinants of adoption. Future research should explore determinants of the successful implementation, adaptation and sustainment of POCUS, which may or may not be the same.

### Limitations and Future Directions

This study has several limitations that should be acknowledged. First, the study population was limited to stakeholders at one academic medical center in the United States. Implementation of complex health interventions, such as POCUS, is highly context-dependent and therefore the generalizability of our results may be limited to similar settings. However, although specific barriers unique to each practice setting must be identified in order to create effective dissemination strategies, we anticipate the dominant themes found in these data, namely cost, efficiency, and clinical impact, will be important in the vast majority of practice settings in the U.S. Another limitation is that we did not interview executive-level managers who are the ultimate decision-makers of a health system. Finally, we chose to forego interviews of patients in this study because there is evidence demonstrating patients generally support POCUS use by clinicians [21,22]. However, gaining a better understanding of the value proposition of POCUS use from the patient perspective, particularly their input on whether this interaction with clinicians does generally increase their engagement and satisfaction as study participants theorized, may be important to helping the health system leaders decide whether to invest in implementation.

To our knowledge this is the first qualitative study to explore clinician- *and* system-level determinants of POCUS adoption in an academic medical center in the U.S. In future work, we hope to determine how differences in contextual factors affect implementation determinants of POCUS applications by extending our work to stakeholders in diverse hospital systems. 

## 5. Conclusions

Determinants perceived to be important to both clinician- and system-level adoption of POCUS use included clinical impact, efficiency, and cost at an U.S. academic medical center. Future studies should focus on how determinants vary across different types of hospital systems so that contextually sensitive implementation strategies may be identified and employed in the pursuit of high-fidelity and sustainable implementation of POCUS applications that optimize patient outcomes.

## Figures and Tables

**Table 1:** Coding Framework using Contextual Domains from the PRISM.

PRISM Contextual Domains	Sub-Domains
**External Environment**	Resources Guidelines (Evidence)
**Internal Environment Setting**	
Organizational Characteristics	Clinician Characteristics Hospital Characteristics
Organizational Perspectives	Clinician Values and Perspectives System Values and Perspectives
Implementation and Sustainability Infrastructure	Workflow (ultrasound equipment availability, information technology infrastructure) Training Credentialing/Quality Assurance Financial Impact

**Table 2:** Participant Demographics.

Stakeholder	Number of Interviewees (*n* = 31)
**Clinicians (*n* = 19)**	
Hospitalists	12
Subspecialists	7
**Hospital Leaders (*n* = 7)**	
Hospital Medicine Clinical Leader	1
Quality and Safety Leaders	2
Clinical Operations Leaders	3
POCUS Committee Leader	1
**Hospital Administrators (*n* = 2)**	
Hospital Medicine Administrator	1
Radiography Administrator	1
**Support Staff (*n* = 3)**	
Information Technologists	2
Radiography Technician	1

**Table 3:** Themes and subthemes.

Themes	PRISM Domain	Subthemes by Level of Stakeholder Adoption	
		Clinician Level	System Level
**Clinical Impact**	**Internal Environment**: Organizational values and perspectives	Potential for both Clinical Benefit and Harm Patient and Physician experience	Quality metrics/Quality assurance Patient satisfaction
**Efficiency**		Learning curve and its effect on efficiency Clinical volume	Length of stay
		“High-value care”	---
**Cost**	**External Environment**: Resources, Policy **Internal Environment**: Implementation and sustainability infrastructure	---	Financial Impact
	**Internal Environment**: Organizational characteristics	---	Who will Pay?

**Table 4:** Participant quotations illustrative of themes.

Themes and Subthemes	Quotation
Clinical Impact	
POCUS has the potential for clinical benefit but also patient harm if quality assurance policy and procedures are not in place.	Clinician A18: “*I think people recognize that making real-time decisions is helpful for patients, because the quicker you can make a decision and effectively administer a treatment to them, the quicker they’re gonna respond, and so I think that’s the name of the game, because we all want to provide the best care for our patients in the most efficient way possible*.” Clinician A28: “*I think the main thing is that if you don’t do it well, and if you don’t have clear guidelines and clear training and then quality control and image review on the backside, then you run the risk of people using POCUS inappropriately or incorrectly interpreting what they’re seeing and then making the wrong decision and leading to harm. I think that’s the biggest downside in my view*.” Hospital Leader G7: “*I think they wanted to make sure that ultrasound wasn’t being used haphazardly for clinical diagnostic purposes, and that we as a community of faculty had the highest level of quality*.”
POCUS has the potential to enhance the patient and clinician experience which is valued by the hospital system	Clinician A15: “*Bringing people to the bedside is really helpful, and I think the patients really like it. They get to talk to the person who’s doing the POCUS, they get to see the images with them, and they get to learn, which, they have all said—I’ve just had positive experiences with my patients who had POCUS done*.” Hospital Leader G2: “*Anything that would help the patient’s experience. Maybe in this example, they have a bedside study instead of having to go to and fro to radiology, that might be avoided and that sort of thing*.”
Efficiency	
Learning Curve	Clinician A5: “*I think just attitude, I guess willingness because it’s one more thing. It takes time. It adds to the busy day. It’s awkward and a little bit stressful for us until you get—getting good and getting fast at it, You have to be excited enough to work through those, climb the learning curve, invest the time and the effort to do it*.”
Clinical Volume	Clinician A8: “*Time definitely plays a role. There are definitely days where—there’s certain patients that I will ultrasound no matter what because I feel like I need to for their clinical care, and then there are some patients where I’m like, I think this might help, and I’m curious to see what it looks like, but it’s not as necessary, and so on busier—really busy days, I just may not get to it. That can definitely influence it, if we’re having a really crazy day*.”
Length of Stay	Hospital Leader G2: “*as you know our issues with capacity, anything that can show to help with that. Then in the end, length of stay also of course, affects money because that bed’s being taken up by somebody else*.”
Cost	
High-value Care	Clinician A27: “*If you take the overall view of value in terms of quality, safety, and experience, for all the good things that promote value, I think point-of-care lung ultrasound for sure ticks the experience bucket because patients really like it. I think providers like it. In terms of safety, as long as it doesn’t harm patients and reduces radiation risk from other modalities, I think it helps there. In terms of quality, if it’s evidence-based and you can make better, faster clinical decisions, then I think it has a potential there. In terms of the cost for the health system, it’s significantly less expensive than a CT scan. It’s probably less expensive than chest X-rays because that comes with people and radiologists and all these things. Again, the payment model dictates some of this, but I think it has the potential to be a high-value care implementation*.”
Financial Impact	Clinician F1: “*If you equate it to financial monetary stuff, that’s the only way you can get anything in medicine approved these days. That’s not me being cynical. That’s just real. You have to show it Reduces costs in some way*.”
Who will pay?	Hospital Leader G6: “*Now you could argue it’s standard of care, but it still doesn’t mean the hospital should pay for it. You can still ask the hospital, and they might pay for it, but they may say, ‘Look, I don’t pay for your stethoscope, do I? No so this is your deal. You pay for it.’*”

## Data Availability

The data presented in this study are available on request from the corresponding author.

## Supplementary Materials

The following are available online at https://www.mdpi.com/article/10.3390/diagnostics11071172/s1, Supplementary Material File S1. Interview guide for Hospitalists.
