# Supplementary material for: Multi-Level Stakeholder Perspectives on Determinants of Point of Care Ultrasound Implementation in a US Academic Medical Center
Source: Diagnostics (Basel). 2021 Jun 28;11(7):1172. doi: 10.3390/diagnostics11071172 (PMC8305030; doi:10.3390/diagnostics11071172)
Supplement: Supplementary file 1 [file diagnostics-11-01172-s001.zip › diagnostics-1197606-supplementary.pdf]

Interview guide for Hospitalists

[*Interviewer: confirm consent. See post card consent]*

We are interviewing stakeholders to understand the determinants of implementation of point of ultrasound. You have been asked to be interviewed because of you may be affected by implementation of POCUS. Interviews will be de-identified, recorded and transcribed. Do you consent to being interviewed?

Thank you for setting this time aside for the interview. Please keep in mind that there are no right or wrong answers to any of the questions I ask you today. Your input and advice are very important as they will help us better understand how best to implement point of care lung ultrasound. If any question is unclear, please ask me to clarify it.

This interview will take about 30 minutes. I would like to record this interview to accurately capture everything that is said. Is that okay? I will now turn on the recorder as we begin the interview. Are you ready?

State date, location, and participant's unique ID number:______ _______________________

1. What is your professional title and job description?

2. What is your practice setting?

3. How many years have you been in practice?

4. What LUS training have you received?

5. How is radiology testing in your hospital different during the COVID pandemic? Is chest imaging ordered at a higher or lower rate in patients with COVID?

6. What is your prior and current experience with lung ultrasound?

7. What diseases do you use it to diagnosis or monitor? (If not, why not?) If **no skip to question 7 and ask about colleagues practice habits**

8. What diseases do you find it most useful for?

9. Can you describe a case in which the lung ultrasound findings changed the working diagnosis or management of one of your patients?

10. Can you describe a case in which the lung ultrasound findings changed the working monitoring or management of a patient with known heart failure?

11. Do you use it in the care of patients with COVID?

12. How is it different from other radiological studies?

13. What are the advantages or value of using lung ultrasound? (probe for types of advantages: to the health system, patient, team, practice flow)

14. What are the disadvantages of using lung ultrasound? (What are you losing by using lung ultrasound?) (probe for types of disadvantages: to the health system, patient, team, practice flow)

15. What do you see as the advantages and disadvantages of chest x-ray vs lung ultrasound?

    In patients with COVID?

16. What aspects of the hospital environment have influenced implementation of lung ultrasound? Can you tell me more about the importance of the influence coming from these systems upon Lung ultrasound?

    a. How has COVID impacted this?

17. What factors from the person operating the ultrasound (like the hospitalist) have influenced implementation of lung ultrasound? Can you tell me more about the importance of the influence coming from these end-user factors upon Lung ultrasound?

    a. How has COVID impacted this?
    b. Are there barriers to LUS use that are specific to COVID?
    c. Have perceptions of the infectious risk changed over the course of the pandemic

18. What would help you to use lung ultrasound?

    a. Who influences this for you (who decides)?

   b. What practice factors influence your decision to use point of care ultrasound?
   c. Do you prefer point of care ultrasound to usual care?
   d. What is your experience uploading images and documenting LUS exams in the EMR?
   e. What frustrations do you have if any about the EMR interface with lung ultrasound use?

19. What role does hospital policy play in the adoption of lung ultrasound by providers?
20. What role do clinical guidelines play in the adoption of lung ultrasound by providers?
21. What level of evidence do you need to adopt lung ultrasound?
22. Who needs to support point of care lung ultrasound implementation for implementation to take place and be sustained?
23. What have been the recent barriers to implementation at UCH from your perspective?
    a. In what ways have they been impacted by caring for COVID patients?
    b. What are the current/remaining barriers to lung ultrasound implementation?
24. What are the potential pitfalls of lung ultrasound implementation from your perspective?
25. What are the potential advantages to the hospital system? What are the potential disadvantages to the hospital system?
26. What role does clinical leadership play in adoption of lung ultrasound?
27. What role does cost and cost savings to the hospital play in the implementation of lung ultrasound?
28. What role do trainees play in the implementation of lung ultrasound
29. What role does reimbursement play in adoption of lung ultrasound by providers?

30. How important is the notion of “high-value care” as a determinant of adoption of lung ultrasound by providers?

31. Can you describe last time you changed your practice to incorporate a new skill?

32. What were the incentives (internal or external) that prompted that change in practice?

33. Who do you see as the other stakeholders in the implementation of point of care ultrasound?

34. Who else would you recommend I speak with?

35. Is there anything else you would like to add or clarify about what we have discussed today?
